# DNA Methylation Changes in Atypical Adenomatous Hyperplasia, Adenocarcinoma *In Situ*, and Lung Adenocarcinoma

**DOI:** 10.1371/journal.pone.0021443

**Published:** 2011-06-23

**Authors:** Suhaida A. Selamat, Janice S. Galler, Amit D. Joshi, M. Nicky Fyfe, Mihaela Campan, Kimberly D. Siegmund, Keith M. Kerr, Ite A. Laird-Offringa

**Affiliations:** 1 Departments of Surgery and of Biochemistry and Molecular Biology, Norris Comprehensive Cancer Center, Keck School of Medicine, University of Southern California, Los Angeles, California, United States of America; 2 Department of Preventive Medicine, Keck School of Medicine, University of Southern California, Los Angeles, California, United States of America; 3 Department of Pathology, Aberdeen Royal Infirmary, University of Aberdeen, Aberdeen, United Kingdom; Vanderbilt University Medical Center, United States of America

## Abstract

**Background:**

Aberrant DNA methylation is common in lung adenocarcinoma, but its timing in the phases of tumor development is largely unknown. Delineating when abnormal DNA methylation arises may provide insight into the natural history of lung adenocarcinoma and the role that DNA methylation alterations play in tumor formation.

**Methodology/Principal Findings:**

We used MethyLight, a sensitive real-time PCR-based quantitative method, to analyze DNA methylation levels at 15 CpG islands that are frequently methylated in lung adenocarcinoma and that we had flagged as potential markers for non-invasive detection. We also used two repeat probes as indicators of global DNA hypomethylation. We examined DNA methylation in 249 tissue samples from 93 subjects, spanning the putative spectrum of peripheral lung adenocarcinoma development: histologically normal adjacent non-tumor lung, atypical adenomatous hyperplasia (AAH), adenocarcinoma *in situ* (AIS, formerly known as bronchioloalveolar carcinoma), and invasive lung adenocarcinoma. Comparison of DNA methylation levels between the lesion types suggests that DNA hypermethylation of distinct loci occurs at different time points during the development of lung adenocarcinoma. DNA methylation at *CDKN2A* ex2 and *PTPRN2* is already significantly elevated in AAH, while CpG islands at *2C35*, *EYA4*, *HOXA1*, *HOXA11*, *NEUROD1*, *NEUROD2* and *TMEFF2* are significantly hypermethylated in AIS. In contrast, hypermethylation at *CDH13*, *CDX2*, *OPCML*, *RASSF1*, *SFRP1* and *TWIST1* and global DNA hypomethylation appear to be present predominantly in invasive cancer.

**Conclusions/Significance:**

The gradual increase in DNA methylation seen for numerous loci in progressively more transformed lesions supports the model in which AAH and AIS are sequential stages in the development of lung adenocarcinoma. The demarcation of DNA methylation changes characteristic for AAH, AIS and adenocarcinoma begins to lay out a possible roadmap for aberrant DNA methylation events in tumor development. In addition, it identifies which DNA methylation changes might be used as molecular markers for the detection of preinvasive lesions.

## Introduction

Lung cancer is the leading cause of cancer-related death in the world, and is estimated to have caused over 1.3 million deaths in 2008 [1], [2]. World wide, smoking accounts for 80% of all lung cancer deaths in males and 50% of those in females [3]. The overall five-year survival of patients with lung cancer is very poor; in the United States it is a dismal 18% despite extensive efforts to improve diagnosis and treatment [4]. Sensitive new visual diagnostic modalities such as low dose spiral computed tomography (LDSCT) show potential to detect much smaller lung lesions than the conventional chest X-ray [5], and reports from studies like the National Lung Screening Trial and the NELSON trial on the effects of LDSCT screening on lung cancer mortality are eagerly awaited [6], [7]. However, even if mortality is reduced, this imaging modality shows limited specificity; while LDSCT can detect stage I cancers, a number of these may not actually progress to late stage cancer [8]. Thus, in order to avoid unnecessary interventions, we must gain better insight into the molecular changes underlying the natural history of lung cancer. Such knowledge could be used to develop additional molecular tests that might complement LDSCT screening, allowing detection of those lesions that would progress to tumors with metastatic potential. The analysis of DNA methylation might provide such a test. Abnormal DNA methylation is an epigenetic change that has been widely observed in all types of cancer including lung cancer [9]–[11]. It consists of the addition of a methyl group to the 5-position of cytosine in the context of a two-base pair palindrome, or CpG dinucleotide. Sensitive molecular assays allow detection of DNA methylation in tumors as well as in patient bodily fluids [11]–[14], and it therefore holds much promise as a possible molecular marker to complement image-based lung cancer screening.

This study focuses on lung adenocarcinoma, a histological subtype of lung cancer that is increasing in many countries [15]–[19], and which currently accounts for at least 37% of all lung cancer in the United States [4]. While smoking remains the predominant cause of lung adenocarcinoma, this histological subtype is also the most common form of lung cancer amongst never smokers, Asians and women [4], [20]. Unlike squamous cell carcinoma, the natural history of lung adenocarcinoma is still poorly understood. Studies suggest that at least some lung adenocarcinomas arise from preneoplastic lesions called atypical adenomatous hyperplasia (AAH), which progress to adenocarcinoma *in situ* (AIS, formerly known as bronchioloalveolar carcinoma or BAC), and eventually develop into invasive cancer [21]–[24]. In 1999, the WHO acknowledged AAH as a putative preneoplastic lesion of lung adenocarcinoma. AAH is now defined as “localized proliferation of mild to moderately atypical cells lining involved alveoli and sometimes respiratory bronchioles, resulting in focal lesions in peripheral alveolated lung, usually less than 5 mm in diameter” [25]. AIS is defined as “a localized small (<3 cm) adenocarcinoma with growth restricted to neoplastic cells along preexisting alveolar structures (lepidic growth), lacking stromal, vascular, or pleural invasion. Papillary or micropapillary patterns and intraalveolar tumor cells are absent.” [24]. AIS is very rarely mucinous. It is associated with a 100% five year post-resection patient survival, and is similar in morphology to high-grade AAH lesions. Both AAH and AIS can be found as incidental findings in the lungs of patients resected for a primary lung tumor, usually adenocarcinoma [21]. However, with the advent of more sensitive radiological imaging, these lesions are now being individually detected using fine section high resolution computed tomography [26], [27]. A number of molecular studies support the existence of an AAH-AIS-adenocarcinoma continuum [9]. LOH events at 9q and 16p, key features of lung cancer, have been reported to occur at similar frequencies in AAH and adenocarcinoma [28], [29], and the mutually exclusive natures of *KRAS* and *EGFR* mutations reported in lung adenocarcinoma are maintained in AAH lesions [30]. Support for a developmental sequence from AAH to adenocarcinoma also comes from conditional oncogenic mouse models for lung adenocarcinoma, in which *KRAS* or *EGFR* genes are activated. In both types of mice, AAH-like lesions are found before the emergence of adenocarcinomas [31], [32].

**Figure 1:**
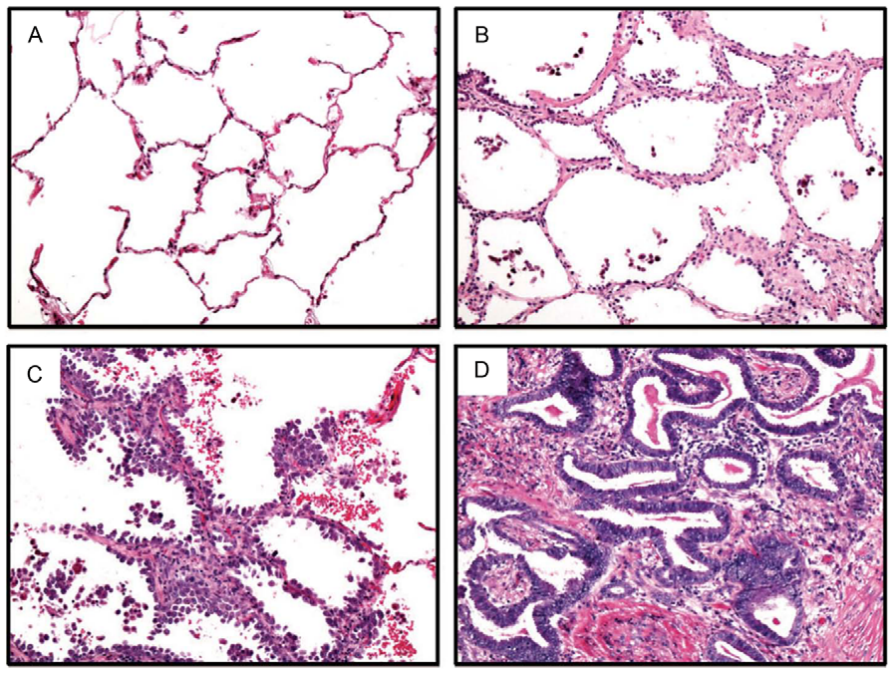
Lung and lesion histology. Haematoxylin and eosin-stained sections of (A) Adjacent non-tumor lung (B) Atypical adenomatous hyperplasia (C) Adenocarcinoma *in situ* (D) Lung adenocarcinoma, all at 100× magnification.

Abnormal DNA methylation has not yet been thoroughly examined in AAH and AIS. Extensive investigation of DNA methylation in AAH has been impeded by the minute size of these lesions and the necessity to use bisulfite conversion. This chemical treatment specifically deaminates unmethylated cytosine to uracil, but not 5-methylated cytosine, thereby embedding DNA methylation information into the DNA sequence. Unfortunately, bisulfite treatment can result in considerable degradation of already scarce genetic material [33]. To date, DNA methylation analysis of AAH has required the use of multiplexed nested methylation-specific polymerase chain reaction (MS-PCR), disallowing quantitative assessment of DNA methylation and limiting the number of genes that can be tested [34], [35]. In this study, we overcame these limitations by using the sensitive technology MethyLight, which consists of real-time PCR of bisulfite-converted DNA, using primers and probes designed to specifically hybridize to methylated regions that retained cytosines [14]. We used MethyLight to successfully quantitatively assess DNA methylation levels at 15 CpG islands prone to hypermethylation in lung adenocarcinoma, and also assessed global hypomethylation by examining DNA methylation of repeated sequences. We examined DNA methylation in tissue samples spanning the putative spectrum of peripheral lung adenocarcinoma development: histologically normal adjacent non-tumor lung from non-lung cancer patients as well as lung cancer patients, AAH, AIS, and invasive lung adenocarcinoma (Figure 1).

Because AAH lesions provide very little DNA, we carefully weighed our choice of the loci to interrogate, selecting 15 loci that constitute strong candidates for non-invasive DNA methylation markers for lung adenocarcinoma detection. From a prescreening of 114 loci, we had previously chosen 28 that were most differentially methylated between tumor and adjacent non-tumor lung for further analysis and had identified 12 of these loci as significantly *hyper*methylated in lung adenocarcinoma [36]. We have subsequently evaluated hundreds more loci (using individual probes, a CpG island microarray, and an Illumina GoldenGate analysis), yielding additional loci highly and frequently methylated in lung adenocarcinoma (unpublished information). From our cumulative data sets, we chose the 15 loci that showed the most promise for development into molecular markers for lung adenocarcinoma (Table S1). These loci are also of interest for the potential biological implications of their DNA methylation. Fourteen of these represented loci that were very frequently and highly methylated: *2C35, CDH13, CDKN2A* ex2, *CDX2, EYA4, HOXA1, HOXA11, NEUROD1, NEUROD2, OPCML, PTPRN2, SFRP1, TMEFF2* and *TWIST1*. We added *RASSF1* because we had observed that, although its methylation frequency is not as high in adenocarcinoma as some other loci [36], [37], it can be methylated in those adenocarcinomas showing little DNA methylation of the other commonly methylated loci, in other words, its DNA methylation profile can be complementary [36]. We have validated these 15 CpG islands as being significantly hypermethylated in lung adenocarcinoma compared to adjacent non-tumor lung in two additional independent sample collections ([36] and unpublished results).

Besides local hypermethylation at CpG islands, global DNA *hypo*methylation is also a hallmark of cancer, and is associated with retrotransposon activation and genomic instability [38]. In order to examine hypomethylation in our analysis, we included two repeat-based DNA methylation probes (*SAT2*-M1 and *ALU*-M2 [39]) in the study. The mean methylation of these two probes has been shown to correlate well with global DNA methylation levels [39]. Thus, our selection of probes was tailored to provide key insights into the occurrence of DNA methylation alterations in putative precursor lesions to lung adenocarcinoma.

## Methods

### Ethics statement

All human tissue samples were paraffin-embedded archival remnants of tissue resected for clinical purposes, and were obtained from Aberdeen University Medical School. The research was approved as exempt from the need to obtain informed consent by the USC IRB (# HS-CG-07-00017) and by the Grampian Research Ethics Committee (study 05/S0801/141). The latter body stipulates that no consents are required from deceased subjects or when de-identified remnants of archival tissue are used. The identities of the subjects were unknown to USC investigators or lab personnel.

### Study subjects

Information on the subjects from whom the samples were procured is provided in Table S2. Because of the archival nature of the samples, and the fact that many patients had long since been deceased, very limited smoking information was available. The distribution of AdjNTL, AAH, AIS and adenocarcinoma tissue samples derived from 63 subjects is described in Tables 1 and 2. AAH lesions are generally quite difficult to find. In order to identify such lesions with more frequency the Department of Pathology, Aberdeen Royal Infirmary, prospectively and specifically examines all surgical lung resection specimens received for AAH and AIS lesions, as well as the index lesion that requires surgical resection. All specimens are inflated per-bronchially with 10% neutral buffered formalin and cut into 1 cm thick parasagittal sections after a 24-hr fixation. All visible lesions of >1 mm diameter are sampled. In addition up to 6 random parenchymal tissue blocks are taken from the lung surrounding but separate from the main lesion, which is most often a primary carcinoma. A minority of lesions is visible to the trained naked eye on such gross examination of the lung slices; most AAH lesions are only detected at microscopy. This approach has provided a high yield of both AAH and AIS lesions over many years [21]. As additional controls we also analyzed 30 independent samples of histologically verified cancer-free lung (MetNTL, see Table S2) from non-lung cancer patients who had been operated for a single pulmonary metastasis from a different organ site (usually colorectal cancer). In total, 249 formalin-fixed paraffin-embedded tissues from 93 subjects were included in the statistical analyses.

**Table 1:** Distribution of AdjNTL, AAH, AIS and adenocarcinoma samples among 63 subjects.

Lesion Type:	AdjNTL	AAH	AIS	Adenocarcinoma
N = 10^1^	+	+		
N = 3^1^	+	+	+	
N = 19	+			+
N = 18	+	+		+
N = 3	+		+	+
N = 10	+	+	+	+
Number of subjects with samples of each type^2^	63	41	16	50

1 13 subjects lacked an adenocarcinoma sample either because it was no longer available (4) or because the patient had a different type of lung cancer (1 mixed adeno/squamous, 5 large cell carcinomas, 2 squamous cell cancers and 1 carcinoid).

2 Total number of subjects from which AdjNTL, AAH, AIS or adenocarcinoma was studied: 63. MetNTL was obtained from an additional 30 subjects.

**Table 2:** Distribution of multiple lesions among the cases.

Number of each type of lesion obtained from a single subject	Subjects with AAH	Subjects with AIS	Subjects with AD
1	23	11	48
2	8	2	2
3	7	0	0
4	2	1	0
5	1	1	0
6	0	0	0
7	0	1	0
*Total subjects*	*41*	*16*	*50*
*Total number of lesions* 1	*73*	*31*	*52*

1 In addition, a single AdjNTL was obtained from each of 63 subjects and a single MetNTL sample was obtained from each of 30 subjects.

### DNA extraction and bisulfite treatment

Each section was hematoxylin stained and evaluated by an experienced pathologist (KMK), who carefully marked the lesions to be retrieved. Slides were manually microdissected under the microscope and DNA was extracted by proteinase K digestion. Microdissected cells were incubated overnight at 50°C in a buffer containing 100 mM TrisHCl (pH 8.0), 10 mM EDTA (pH 8.0), 1 mg/ml proteinase K, and 0.05 mg/mL tRNA. Extracted DNA was bisulfite converted using Zymo EZ DNA Methylation kit (Zymo Research, Orange, CA) with a modification to the protocol in which samples were cycled at 90°C for 30 seconds and then 50°C for one hour, for up to 16 hours total. Bisulfite-treated DNA was subjected to quality control tests for DNA amount and bisulfite conversion [40]. DNA levels were determined by a bisulfite conversion-independent *ALU* reaction (*ALU*-C4), consisting of a primer/probe set lacking CpGs [40]. A conservative cutoff was set at C_t_ (threshold cycle) ≤22 after extensive analyses comparing data with a cutoff of *ALU* C_t_ ≤20 with that of C_t_ ≤22 showed no statistically significant difference in percentage methylated reference (PMR) values (see below) between the two (data not shown). In addition, a previous study demonstrated that samples with C_t_ values ≤24 still yielded reliable results [41]. Four independent AAH samples with *ALU* C_t_ values >22 were thus excluded.

### DNA methylation analysis

Bisulfite-treated DNA was analyzed by MethyLight as described [42]. Primer and probe sequences are listed in Table S1. Locus *2C35* was identified by restriction landmark genomic sequencing to be highly methylated in non-small cell lung cancer [43] as well as other types of cancer [44]. The *CDKN2A* ex2 primer/probe set detects highly significant hypermethylation in a CpG island in exon 2 of *CDKN2A* in lung adenocarcinoma vs. adjacent non-tumor lung, showing more highly significant differences than probes for upstream CpG islands [36]. The *OPCML* primer/probe set also targets the CpG island of the adjacent and closely related family member HNT [36]. The *SAT2* and *ALU* probes (*SAT2*-M1 and *ALU*-M2) DNA methylation values were averaged and used as an indicator for global DNA methylation levels [39]. *ALU*-M2 is *distinct* from the *ALU*-C4 probe that hybridizes to a methylation-independent (CpG-less) region of *ALU* repeats and that was used for input DNA normalization [40]. Genomic DNA which was exhaustively enzymatically methylated by three consecutive M.*Sss*I treatments was used as a reference sample to generate standard curves. MethyLight data is represented as the percentage methylated reference (PMR), which is defined by the *GENE*: *ALU*-C4 ratio of a sample, divided by the *GENE*: *ALU*-C4 ratio of M.*Sss*I-treated reference DNA [40]. While it is rare, occasionally PMR values of more than 100 can be observed, indicating that the reference DNA might not be fully methylated at a particular site. The same batch of reference DNA was used throughout this study to avoid any bias.

### Statistical analyses

We included a total of 249 tissue samples from 93 subjects in the analysis. Our primary statistical analysis was to compare the DNA methylation values between groups of different lesion types using generalized estimating equations (GEE [45]). GEE is a regression approach that allows us to use all lesions of the same type from the same individual in the analysis, while properly accounting for the possible within-individual correlation in DNA methylation values. We first verified that the data satisfy the assumption that the average DNA methylation value was the same in lesions from patients with one lesion compared to patients with multiple lesions of the same type (data not shown). For each marker, two groups were then compared by regressing the rank of the PMR values on an indicator variable for group membership. The rank transformation was used to address skewness in the PMR value when testing for differences in group means, ranking all 249 samples before proceeding with the pair-wise group comparisons. Hypothesis testing used robust variance estimates under an independence working correlation structure. All testing was performed at the 5% significance level.

To identify in which lesion type, AAH, AIS, or adenocarcinoma, the markers first showed a difference in average DNA methylation value, we performed a series of univariate tests, comparing DNA methylation values between pairs of histologies: AdjNTL vs. AAH, AAH vs. AIS, and AIS vs. adenocarcinoma. To account for conducting three tests for each marker (multiple testing), we applied a Bonferroni correction to determine statistical significance, requiring a cutoff of p<0.017 ( = 0.05/3 tests) for statistical significance. Markers were classified into the categories “early”, “intermediate”, or “late”, depending on the pairwise comparison that yielded the first increase in average DNA methylation value that both achieved statistical significance, and showed a group median of >1 on the raw PMR scale. The PMR scale runs from 0 to 100 (100 indicates complete methylation compared to enzymatically methylated human DNA); a >1 PMR cut-off was chosen to minimize undue emphasis on very low levels of DNA methylation that are not likely to be biologically significant. Following this analysis, we investigated the potential for a “field defect” in the lung by comparing DNA methylation values in AdjNTL with MetNTL. As none of the 15 hypermethylation markers or the hypomethylation measure had been compared previously between these two tissue types, we controlled for multiple testing by requiring a Bonferroni-corrected p-value (p<0.0031 = 0.05/16 tests) to declare statistical significance.

We performed a cluster analysis to see if we could identify any subgroups within AAH lesions. Using the 15 hypermethylation loci, we applied partitioning around medoids (PAM) [46], using silhouette width to select the number of clusters. For all markers, DNA methylation values were compared between the two identified clusters using GEE and a Bonferroni cutoff of p<0.0033 (15 tests). The same methods were applied to compare AAH lesions based on histologic grade: high grade (HG) and low grade (LG).

To examine the potential effects of clinical variables on the analysis, we used ANOVA (for age and packyears) and the Chi-square test (for gender and known smoking status) to examine whether these variables differed significantly between sample types (MetNTL, AdjNTL, AAH, AIS, adenocarcinoma). Statistical analyses were performed using STATA version 10, Prism 5, and R.2.10.0.

## Results

### DNA methylation levels across the AdjNTL-AAH-AIS-adenocarcinoma spectrum

We used a comprehensive collection of tissues encompassing adjacent non-tumor lung (AdjNTL), the putative adenocarcinoma precursor lesions AAH and AIS, as well as synchronous adenocarcinoma (Tables 1 and 2). Since AdjNTL from lung cancer patients might show DNA methylation “field defects” and general molecular changes arising from environmental exposures such as tobacco smoke [47], we included adjacent lung tissue from resections of 30 subjects with single pulmonary metastases from non-lung primary cancers (MetNTL) in the study. Our sample collection also included cases in which multiple AAH and AIS lesions were obtained from a single subject (Table 2), which allowed evaluation of the spectrum of DNA methylation changes within individuals. Each of the AAH and AIS specimens was pathologically confirmed to be an isolated lesion separate from any other lesions in the same lung.

We had previously found all 15 CpG islands to be highly significantly methylated in lung adenocarcinoma compared to AdjNTL (tissues derived from lung cancer patients from the Los Angeles area, the East coast of the United States, and Ontario, Canada ([36] and unpublished data). Here, we confirmed these findings, observing highly significant DNA hypermethylation in adenocarcinoma vs. AdjNTL for all 15 hypermethylation loci (all p<1×10^−5^, Table 3) in samples originating from the United Kingdom. This indicates that lung adenocarcinoma samples from a variety of geographic areas can exhibit similar hypermethylation profiles.

**Table 3:** Median Percentage Methylated Reference (PMR) and pair-wise comparison p-values between each tissue type.

	Median PMRs					p-values for pair-wise comparisons of tissue types^1^					
Locus	MetNT (n = 30)	AdjNT (n = 63)	AHH (n = 73)	AIS (n = 31)	AD^2^ (n = 52)	MetNT vs. AdjNTL	AdjNTL vs. AAH	AAH vs. AIS	AIS vs. AD	AdjNTL vs. AD	Designation
*BH p-value threshold*						*0.0031*	*0.017*	*0.017*	*0.017*	*0.05*	
*CDKN2A EX2*	4.5	5.0	11	19	21	0.42	**2.6E-11**	0.045	0.90	**<2.1E-14**	Early
*PTPRN2*	3.7	1.1	2.8	8.8	19	**7.7E-5**	**2.1E-3**	0.023	**5.5E-3**	**<2.1E-14**	Early
*2C35*	0.53	0.60	1.1	11	24	0.54	0.18	**7.2E-4**	0.032	**<2.1E-14**	Interm
*EYA4*	2.0	1.6	0.41	3.1	16	0.94	0.092	**7.0E-4**	**1.5E-4**	**1.3E-11**	Interm
*HOXA1*	<0.01	0.015	0.12	4.6	21	0.15	0.16	**8.7E-5**	0.037	**<2.1E-14**	Interm
*HOXA11*	1.5	0.92	1.3	7.8	19	0.014	0.23	**6.7E-8**	**1.4E-4**	**<2.1E-14**	Interm
*NEUROD1*	0.29	0.17	0.60	3.9	13	0.18	0.014	**0.011**	**3.1E-3**	**<2.1E-14**	Interm
*NEUROD2*	0.78	1.3	1.2	4.0	12	0.0056	0.71	**0.016**	**7.1E-3**	**<2.1E-14**	Interm
*TMEFF2*	6.1	4.9	6.1	19	18	0.089	0.19	**1.9E-9**	0.23	**8.9E-7**	Interm
*CDH13*	<0.01	<0.01	0	<0.01	1.3	0.89	0.039	4.8E-3	**5.5E-10**	**2.1E-14**	Late
*CDX2*	1.3	0.46	0.53	1.6	10	0.17	0.94	0.061	**6.1E-3**	**<2.1E-14**	Late
*OPCML/* *HNT* 3	0.32	0.028	0	0.29	5.3	0.097	0.067	1.5E-3	**3.0E-4**	**<2.1E-14**	Late
*RASSF1*	0.44	0.12	<0.01	0.23	8.9	8.0E-4	0.0090	9.0E-3	0.026	**8.5E-6**	Late
*SFRP1*	0.29	0.44	0.15	0.29	8.0	0.27	0.071	0.52	**6.9E-12**	**2.9E-14**	Late
*TWIST1*	0.010	<0.01	0	0.12	16	0.40	4.4E-3	4.2E-3	**9.9E-3**	**<2.1E-14**	Late
Mean Repeats	80	71	72	75	46	0.076	0.12	0.99	**1.0E-9**	**2.4E-9**	Late

1 p-values are from GEE analysis. Designations of “early” “intermediate” and “late” are based on statistically significant p-values with the restriction that any locus designated as hypermethylated have a median PMR value of ≥1 (bolded). This was done to minimize attributing significance to biologically meaningless differences.

2 AD = adenocarcinoma.

3 The OPCML/HNT primer/probe set recognizes two adjacent CpG islands for the homologous OPCML and HNT genes.

**Figure 2:**
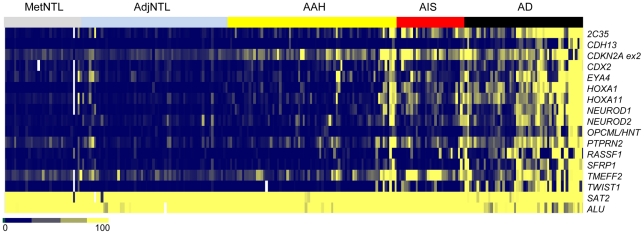
Heatmap of DNA methylation levels of 15 loci and repeats in all tissue types. Loci are arranged in alphabetical order. Dark blue indicates very low levels of DNA methylation, yellow indicates high levels of DNA methylation, and missing values are indicated in white. The type of sample is indicated at the top.

**Figure 3:**
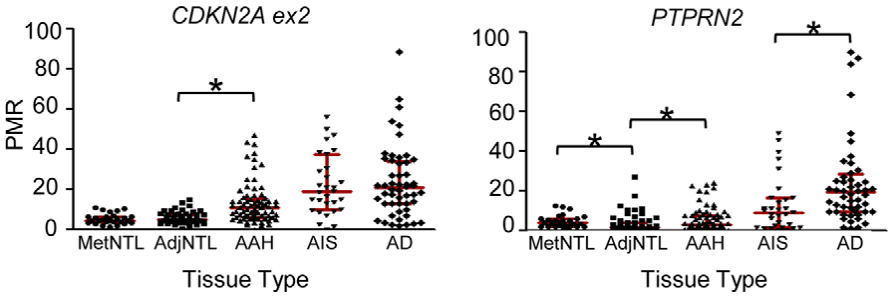
“Early” DNA methylation changes: scatterplots of loci significantly hypermethylated in AAH lesions compared to AdjNTL. p-values were calculated by GEE, with a Bonferroni cutoff of p<0.017 (see Methods). Statistically significant differences are marked with an asterisk. Interquartile ranges are marked with red bars.

**Figure 4:**
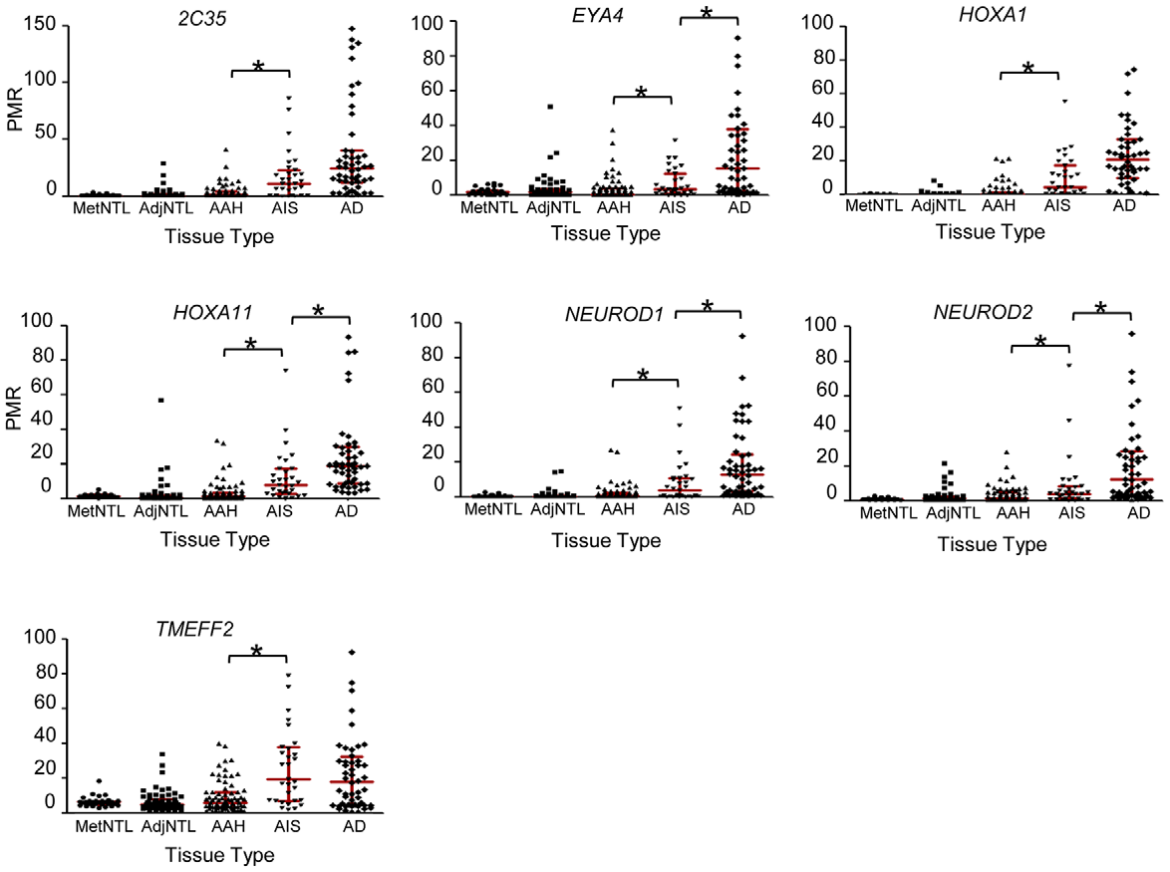
“Intermediate” DNA methylation changes: scatterplots of loci significantly hypermethylated in AIS lesions compared to AAH. p-values were calculated by GEE, with a Bonferroni cutoff of p<0.017 (see Methods). Statistically significant differences are marked with an asterisk. Interquartile ranges are marked with red bars.

**Figure 5:**
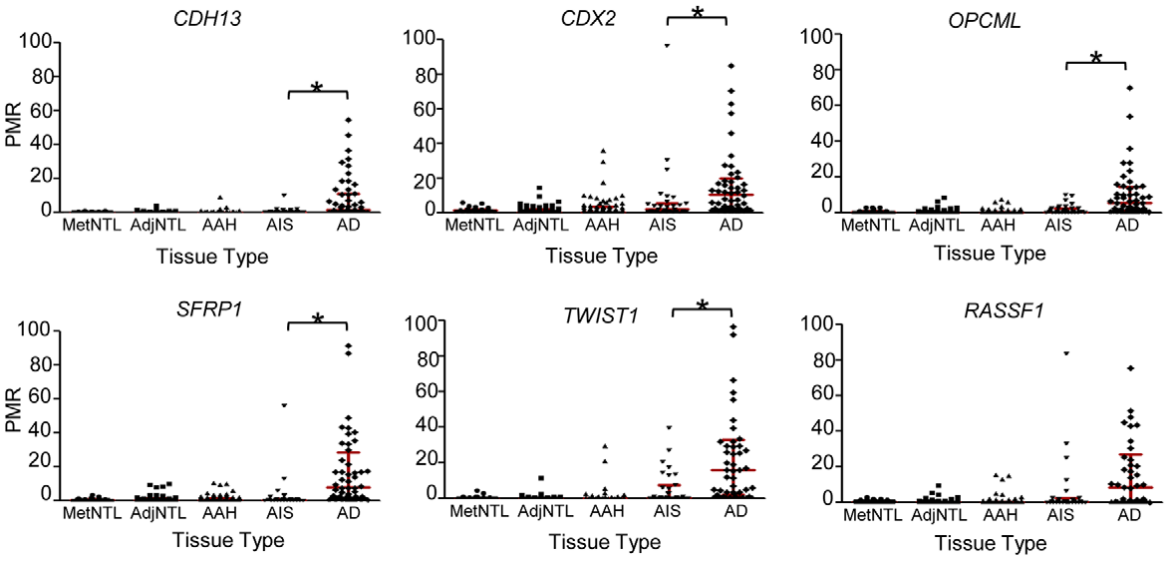
“Late” DNA methylation changes: scatterplots of loci significantly hypermethylated in adenocarcinoma compared to AIS. p-values were calculated by GEE, with a Bonferroni cutoff of p<0.017 (see Methods). Statistically significant differences are marked with an asterisk. Interquartile ranges are marked with red bars. RASSF1 was included in the figure because hypermethylation is clearly present increased adenocarcinoma, although the AIS vs. adenocarcinoma comparison did not reach statistical significance (p = 0.026, see Table 3).

**Figure 6:**
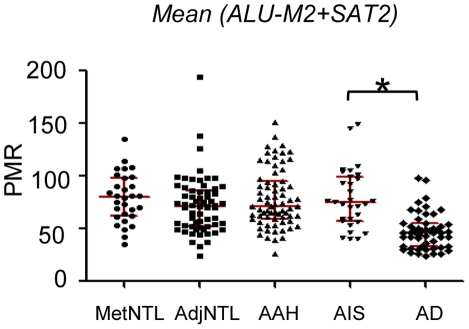
Global DNA methylation levels in AAH, AIS, and lung adenocarcinoma. The average of *ALU*-M2 and *SAT2*-M1 probes were used as indicators of global DNA methylation. p-values were calculated by GEE, with a Bonferroni cutoff of p<0.017 (see Methods). Statistically significant differences are marked with an asterisk. Interquartile ranges are marked with red bars.

We next determined whether these DNA methylation changes are present in the presumptive precursor stages of the disease, AAH and AIS (Figure 2 and Table 3). Markers were classified into the categories “early”, “intermediate”, or “late”, depending on the pairwise comparison that yielded the first increase in average DNA methylation value that both achieved statistical significance, and showed a group median of >1 on the raw PMR scale (Table 3). According to these criteria, *CDKN2A* ex2 and *PTPRN2* were designated as “early” loci, with statistically significantly higher DNA methylation in AAH than in AdjNTL (Figure 3). *PTPRN2* showed a further significant increase in DNA methylation in adenocarcinoma vs. AIS (the increase from AAH to AIS did not meet our multiple comparisons threshold). Seven loci, *2C35, EYA4, HOXA1, HOXA11, NEUROD1, NEUROD2* and *TMEFF2*, were designated as “intermediate”, or characteristic for AIS (Figure 4). Significant DNA hypermethylation of these loci was observed in AIS compared to AAH, and for four of these loci, DNA methylation levels further increased significantly in adenocarcinoma compared to AIS. Five remaining loci, *CDH13, CDX2, OPCML, SFRP1* and *TWIST1* were designated as “late” loci; significantly elevated DNA hypermethylation was only detected in adenocarcinoma, as compared with AIS (Figure 5). *RASSF1* hypermethylation approached significance but did not meet our multiple comparisons cut-off in the AIS to adenocarcinoma comparison. However, RASSF1 was highly significantly hypermethylated in adenocarcinoma vs. AdjNTL, and the scatterplot supports the notion that *RASSF1* hypermethylation is a late event (Figure 5). Significant DNA hypermethylation of these six loci would therefore appear to be associated with invasive lung adenocarcinoma. Examination of the mean of the two repeat probes as an indicator of global DNA hypomethylation showed highly significant hypomethylation only in the AIS to adenocarcinoma comparison (Figure 6) suggesting that global DNA hypomethylation may be a late event in lung adenocarcinoma development.

Baseline DNA methylation levels in AdjNTL were in general low, however, modest methylation was observed for several of the 15 DNA hypermethylation markers (Table 3). To determine whether these potentially elevated DNA methylation levels could be an indication of a “field defect”, we compared DNA methylation levels of the 15 hypermethylation probes and the global DNA methylation measure in AdjNTL vs. MetNTL. Only one hypermethylation locus met the criterion for a statistically significant difference in methylation between the two tissue types: *PTPRN2* (Table 3). DNA methylation levels for *PTPRN2* were *lower* in AdjNTL compared to MetNTL (median PMR of 1 vs. 4), not higher. This difference is difficult to discern from Figure 3 due to low variation in PMR values (interquartile ranges of 0.4–3.3 for AdjNTL and 2.0–5.5 for MetNTL) and the scale on the vertical axis. *PTPRN2* also showed significantly increased DNA methylation from AIS to adenocarcinoma. Thus, we did not find elevated DNA methylation in AdjNTL compared to MetNTL for any locus, nor did we observe any significant difference in global hypomethylation (Table 3, bottom row).

With the limited smoking information we had, we examined whether smoking status (current or past) or packyears of smoking were associated with DNA methylation levels seen in AdjNTL, and might explain the variability seen in baseline DNA methylation levels. We observed no significant differences (data not shown).

### Analysis of DNA methylation in preneoplastic lesions

It has been proposed that not all AAH lesions progress to cancer. If true, some AAH could show molecular changes indicative of their propensity to progress. In order to assess the existence of any sub-groups of preneoplastic lesions differing in DNA methylation profiles, we examined the relationship of the samples and the 15 DNA hypermethylation probes using partitioning around medoids (PAM). We observed two distinct clusters of 68 and 5 samples. In the latter group, the five AAH lesions from four individuals had statistically significantly higher DNA methylation levels for *2C35, CDKN2A* ex2, *CDX2, HOXA1, NEUROD1, TMEFF2* and *TWIST1* than the remaining 68 samples (all p<0.003).

AAH lesions are sometimes divided into high grade (HG) and low grade (LG) based on histology. However, this distinction can be rather subjective. The grade determination did not correlate with our delineation of the two AAH clusters. We compared PMR values from AAH lesions histologically denoted as high-grade (HG, n = 11) to low-grade (LG, n = 45) lesions and found no statistically significant differential DNA methylation between the two histologies after multiple comparison correction (Table S3).

## Discussion

**Figure 7:**
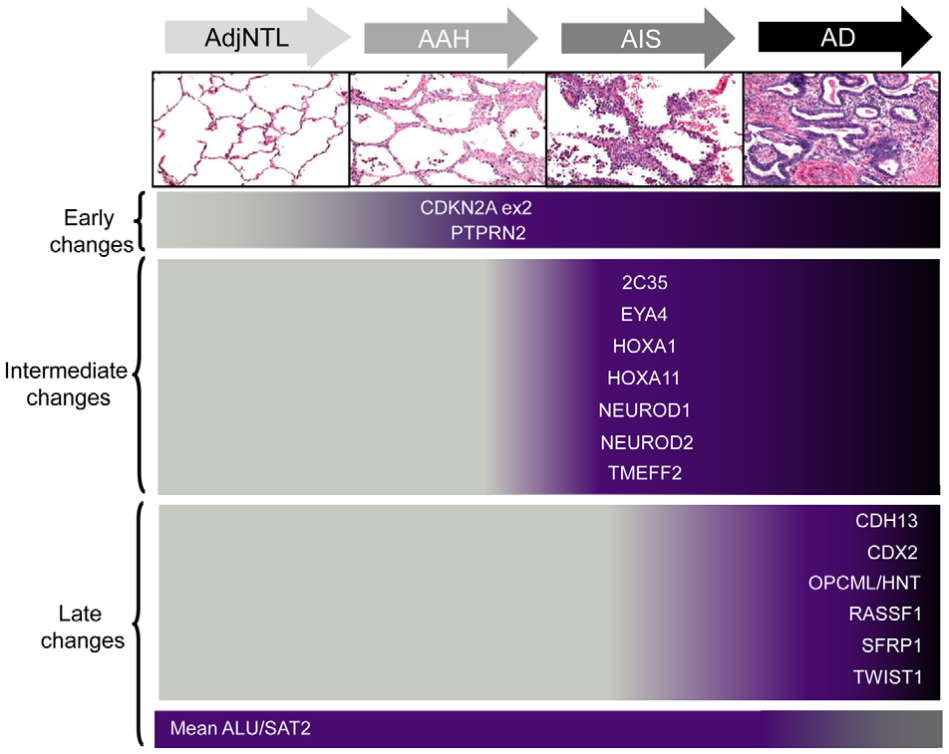
Summary of DNA methylation changes in AAH, AIS, and lung adenocarcinoma. The putative sequence of DNA hypermethylation events is indicated by the color shading and position of locus names. Dark shading indicates hypermethylation. Global DNA hypomethylation is only significantly altered in the AIS to adenocarcinoma comparison, though it appears to occur sporadically even in histologically normal tissue.

Our observation that distinct loci show DNA hypermethylation at different stages of the putative adenocarcinoma development sequence and that the number of methylated loci and DNA methylation levels are generally higher in each progressive stage, support a model in which AAH and AIS are precursor stages of at least a subset of lung adenocarcinomas. The data indicate that distinct epigenetic events occur with the transition to hyperplasia, carcinoma *in situ* and finally invasive cancer (summarized in Figure 7) and imply a model similar to that for the development of colorectal and breast cancers [48]–[51]. Our quantitative observations build on previous reports of increased DNA methylation frequency in AAH compared to adjacent non-tumor tissue [34], [35] and suggest that this trend continues in the AIS to adenocarcinoma continuum. A longitudinal study, in which lesions are studied over time in the same individual, would be the best way to study the natural history of cancer, but this is very difficult to do for peripheral lung cancer given the small size and inaccessibility of preinvasive lesions. Because our study was cross-sectional, comparing individual lesions from a collection of patients, any temporal interpretations should be treated with caution; the results could be affected by confounding factors such as age, gender and smoking history. Examination of gender and age showed no significant differences between AdjNTL, AAH, AIS and adenocarcinoma groups, nor did we find a relationship between smoking status (current of former) or packyears and DNA methylation levels. However, the number of subjects for which any smoking information was available was small (n = 19). To further support our findings, we therefore also examined two subsets of samples from our collection: the samples from the 10 subjects from whom AdjNTL, AAH, AIS, adenocarcinoma were all available (top row, Table 1), and the collection of samples obtained from 16 confirmed current or previous smokers with at least 20 packyears or more of smoking. While the two subsets had a much smaller sample size and therefore had less power than the full collection, we observed that the hypomethylation measure and the majority of hypermethylation loci (10/15) showed similar changes in median PMR and would classify to the same category (early, intermediate or late) in both subsets, either through statistical significance or trending to statistical significance (data not shown). This suggests that our observations are robust and not the result of confounding factors.

Of the 15 loci we studied, the CpG islands of *CDKN2A* ex2 and *PTPRN2* are the only two that we found to be significantly hypermethylated in AAH lesions compared to adjacent non-tumor lung. Frequent deletions and mutations of *CDKN2A* (a negative regulator of cell cycle, also known as p16) in lung cancer were first observed in 1994 [52] and hypermethylation and silencing was subsequently observed to occur in substantial numbers of cancers carrying an intact gene [53]. Inactivation of *CDKN2A* by DNA hypermethylation is now thought to be one of the earliest events during lung cancer development ([54] and references therein) and is observed in hyperplasia and carcinoma *in situ* [34], [55], [56]. We focused on the exon 2 CpG island of the gene because our previous study of *CDKN2A* DNA methylation showed that it was more highly significantly associated with cancer compared to adjacent non-tumor lung than the promoter CpG island. However, it should be noted that some cancer cell line data suggests that *CDKN2A* exon 2 DNA methylation is not necessarily associated with gene silencing [57]. Methyl-binding protein MeCP2 has been shown to associate with methylated *CDKN2A* exon 2, but the biological significance of this modification for cancer progression remains to be clarified [57]. The functional consequences of *CDKN2A* exon 2 DNA methylation in tumor samples merits further investigation. Interestingly, *CDKN2A* hypermethylation at the promoter CpG island been associated with progression of stage 1 lung cancer [58], suggesting the importance of continued inactivation of this gene during progression.

Little is known about the function of *PTPRN2*, a receptor type protein tyrosine phosphatase (PTP) that is a major autoantigen in insulin-dependent diabetes mellitus [59] and that is also expressed in the cerebellum and other parts of the nervous system [60]. Because PTPs dephosphorylate proteins, many of these enzymes are implicated in the negative regulation of cell growth, differentiation and oncogenic transformation [61]. A variety of PTPs have been shown to be mutated in colorectal cancer [62] and PTP receptor-type D was identified as mutated and inactivated in lung adenocarcinoma [63]. We have found *PTPRN2* to be frequently methylated in adenocarcinoma and squamous cell cancer of the lung, in at least two independent sample sets for both histological subtypes ([64] and unpublished results). However, functional studies on the potential role of this protein in any type of cancer remain to be done.

We observed significant DNA hypermethylation in AIS compared to AAH for seven loci: *2C35, EYA4, HOXA1, HOXA11, NEUROD1, NEUROD2* and *TEMFF2*. *2C35* was identified through restriction landmark genomic scanning to be hypermethylated in lung cancer [43] as well as in primitive neuroectodermal tumors, gliomas and colon cancer, and these observations were the basis for the design of MethyLight probe/primer set and our examination of this locus in lung cancer. The CpG island does not overlap with a known gene, although it overlaps with an uncharacterized expressed sequence tag (DA773580 [65]). The nearest identified gene, located 20 kb downstream, is *PTF1A*, pancreas specific transcription factor 1a, a helix-loop-helix transcription factor promoting acinar differentiation in the pancreas and showing loss of function in pancreatic cancer [66]. To date no role of *PTF1A* in lung cancer has been reported. Thus, the biological relevance of DNA methylation at *2C35* remains to be investigated. One possibility is that this locus carries an enhancer that might normally drive the expression of one or more distant genes; in human H1 embryonic stem cells the region containing *2C35* shows histone 3 lysine 4 mono-methylation, a mark that is associated with enhancers and regions downstream of transcription start sites (data from the Bernstein lab at the Broad Institute, [67]). *EYA4*, the human homologue for eyes-absent 4 from Drosophila, is a tyrosine phosphatase that targets histone H2AX and plays a role in recruiting the DNA repair machinery to DNA. The gene is inactivated by DNA methylation in Barrett's esophagus and esophageal adenocarcinoma [68]. We found significant DNA hypermethylation of both *HOXA1* and *HOXA11*, which lie about 90 kilobases apart at opposite ends of the *HOXA* cluster, in AIS. *HOX* genes have been reported to be coordinately hypermethylated in lung cancer, particularly adenocarcinoma [69], [70]. In breast cancer, *HOXA1* was identified as a frequently methylated, and in an analysis similar to ours, was found to be significantly hypermethylated in atypical ductal hyperplasia (ADH) relative to normal breast, and ductal carcinoma in situ (DCIS) relative to ADH [50]. However, no multiple comparisons correction was applied in the latter study; using such a correction *HOXA1* is only significantly hypermethylated in DCIS vs. ADH, which is very similar to our finding of significant hypermethylation in AIS compared to AAH. *TMEFF2*, a transmembrane protein with EGF-like and two follistatin-like domains (also known as hyperplastic polyposis protein (*HPP1*) and tomoregulin), was found to be similarly hypermethylated in DCIS in the breast cancer study. *TMEFF2* had previously been reported to be methylated in lung adenocarcinoma [71], and inactivation of DNA methyltransferase 1 in a breast cancer cell line reactivates methylated *TMEFF2* [72], suggesting its DNA methylation leads to silencing. We thus identified two loci, *HOXA1* and *TMEFF2*, that appear to have an “intermediate” role in cancer development in the lung as well as the breast. *NEUROD1* and *2* were identified by us as highly methylated in lung adenocarcinoma compared to AdjNTL. *NEUROD1* DNA methylation has been observed in diffuse large B-cell lymphoma [73] and breast cancer where it was associated with a ten-fold more likely response to neoadjuvant therapy in estrogen receptor-negative cancers [74]. It is intriguing that just like *PTPRN2*, *NEUROD* proteins appear to be involved both in diabetes mellitus and cerebellar development [75].

*CDH13, CDX2, OPCML, SFRP1* and *TWIST1* do not show significant hypermethylation in AAH or AIS, and instead are only significantly hypermethylated in invasive adenocarcinoma. Inactivation or hypermethylation of many of the latter genes has been linked to poor prognosis or metastasis, agreeing with a potential role in the development of invasive cancer. *CDH13* or heart cadherin, encoding and adhesion molecule, was identified as DNA hypermethylated in lung cancer in 1998 [76], a finding that was substantiated by many studies (e.g. [36], [77]–[80]). *CDH13* DNA methylation has been found to be associated with stage IV disease [81], poor prognosis [82], and tumorigenicity of xenografts in nude mice [83]. In a silica-induced lung cancer animal model, *CDH13* DNA methylation was seen in invasive but not preinvasive lung cancer [84], and in an analysis of stage I lung cancer patients, it was observed to be associated with recurrent cancer [58]. Thus, loss of *CDH13* may be linked to the altered adhesive properties that allow cells to become invasive. Likewise, *OPCML*, an opioid receptor and putative tumor suppressor thought to play a role in adhesion [85], appears to become DNA methylated late, showing hypermethylation mainly in adenocarcinoma. Silencing of *OPCML* has been implicated in metastasis of gastric cancer [86]. *SFRP1*, encoding secreted frizzled-related protein, a *WNT* signaling pathway antagonist, is another “late” locus. *SFRP1* was previously examined in AAH lesions, and was found to be DNA methylated in 11–14% of AAH lesions [35]. In our hands, DNA methylation of *SFRP1* in AAH was even less frequent, and we see little DNA methylation in AIS. Like Licchesi *et al*., we observe dramatic hypermethylation of *SFRP1* in adenocarcinoma (Figure 5), suggesting that the DNA methylation of this gene may be a key change associated with invasion. Transcriptional silencing of *SFRP1* by DNA methylation and loss of heterozygosity in lung cancer have been documented, supporting a role for this gene as a tumor suppressor [87], [88], and *SFRP1* hypermethylation was found to be associated with lymph node metastasis and progression [88]. The silencing of *SFRP1* is especially of interest since the *WNT* pathway was recently implicated in lung adenocarcinoma metastasis [89]. *TWIST1*, encoding a helix-loop-helix transcription factor, was identified as DNA methylated in lung cancer based on a genome-wide screen for genes reactivated in lung cancer cell lines by 5-aza-2′deoxycitidine, a DNA methyltransferase inhibitor [90]. The locus has also been found to be highly methylated in metastatic breast cancer [91]. Intriguingly, *overexpression* of *TWIST1* has been linked to invasion and metastasis in hepatocellular carcinoma and oesophageal cancer [92], [93]. These observations suggest that further studies of *TWIST1* to clarify its role in invasion and metastasis are warranted. Of the genes we characterized as becoming DNA methylated as AIS becomes invasive, *CDX2* has been least well studied. In colorectal cancer, its DNA methylation appears to cause silencing and seems to be associated with advanced stage disease and poor prognosis [94], [95]. In the study of stage I lung cancer patients mentioned above, DNA methylation of *RASSF1*, a ras-associated putative tumor suppressor, was also found to be associated with recurrence [58]. Numerous groups have reported *RASSF1* DNA methylation in lung cancer [37], [96]–[98], and methylation of this gene has been associated with poor prognosis [96], [99] and later stage cancer [100]. The latter observations would appear to be in agreement with our characterization of *RASSF1* DNA methylation as associated with the transition from *in situ* cancer to invasive cancer. While we observed occasional hypermethylation of *RASSF1* in both AAH and AIS, the frequency in these preinvasive lesions was low, and DNA methylation levels were also low. The DNA methylation frequency we observed in the tumors was comparable to that found by us and others [37], [96]–[98]. It is interesting therefore that *RASSF1* DNA methylation has been found in the sputum of smokers prior to the detection of overt lung cancer [101]. In the only other analysis of *RASSF1* DNA methylation in AAH lesions [34], methylation of the locus was reported in almost 30% of AAH, a frequency that approaches that reported for tumors. The lower frequency we observe in AAH in our study might be attributable to our use of a quantitative technique to measure DNA methylation, and to the fact that our probe/primer set detects methylation of 6 CpGs in the amplicon, thus providing a more strict measurement of hypermethylation.

To obtain an indicator for the timing of *hypo*methylation with respect to lung adenocarcinoma development, we used the mean of two repeat-based probes used as measures for global DNA methylation [39]. To our knowledge, global DNA methylation has not been previously investigated in the putative preinvasive stages of lung adenocarcinoma. We observe highly significant hypomethylation only in adenocarcinoma, suggesting that pervasive global hypomethylation is a later event than hypermethylation. However, it should be noted that there is quite a wide spread of global DNA methylation levels in all of the sample types we tested (Figure 6). A recent study of global hypomethylation in stage 1 lung cancer found it to be significantly associated with stage IB *vs*. IA, larger tumors and less differentiated morphology [102], indicating that it may indeed be a later rather than earlier event.

As a comparison for the studied (pre)malignant lesions, we examined two types of histologically normal lung tissue, AdjNTL and MetNTL. We observed no increased DNA methylation of the 15 loci in AdjNTL compared to MetNTL. This is especially telling, since the median age of the MetNTL subjects was slightly younger (Table S2), and increased DNA methylation with age has been reported [103]; if observed, a slightly higher DNA methylation in AdjNTL could have been attributed to the small age difference. The lack of higher DNA methylation in AdjNTL strongly suggests that there is no field defect for these loci, at least when compared to histologically normal lung from patients with a metastasis to the lung. We did observe significantly higher DNA methylation of *PTPRN2* in MetNTL. One possible explanation is that something is different about *PTPRN2* in the cases from which MetNTL was obtained. We have no basis for assuming that *PTPRN2* values for AdjNTL are not representative and for some reason were abnormally low.

While we did not find increased DNA methylation in any of our 15 DNA hypermethylation loci between high-grade or low-grade AAH, in an unsupervised analysis we identified a small group of five AAH lesions that showed significantly higher levels of DNA methylation in seven loci: *2C35, CDKN2A* ex2, *CDX2, HOXA1, NEUROD1, TMEFF2* and *TWIST1*. Whether this elevated DNA is somehow related to the propensity to progress will require further studies, but it is notable that four of these loci are ones that were designated “intermediate” for increased DNA methylation in AIS. The four patients carrying the AAH that were more highly methylated did not consistently show unusually high DNA methylation in their other lesions, confirming that lesions found in patients are independent. The small number of lesions that clusters separately from the main group of AAH would be in keeping with a model in which the majority of AAH lesions may never progress. The five AAH lesions in the small cluster were a mixture of HG and LG lesions, again indicating no link between hypermethylation and grade designation in AAH. One could wonder whether the 5 separately clustering AAH samples were the ones driving the designation of *CDKN2A* ex2 as an “early” hypermethylation change, since they exhibited higher levels of DNA methylation of this locus than other AAH samples. However, when we reanalyzed the data set with the omission of these five samples the difference in DNA methylation of *CDKN2A* ex2 between AdjNTL and AAH was still highly significant (p<0.000001), supporting its designation as an “early” DNA methylation event occurring as hyperplasia develops in the peripheral lung.

Of interest was the observation that the patient for whom two AAH lesions partitioned to the small cluster had 7 AIS lesions. Comparison of DNA hypermethylation levels between single AAH or AIS lesions and those from subjects in whom two or more lesions were found showed no statistical differences in PMR levels for any of the CpG islands, and the distribution of PMR values was comparable to that of the single AAHs or AISs (not shown). Thus, it would not appear that persons with many AAH or AIS lesions show generally increased DNA methylation levels in these lesions.

For those loci for which it is unknown whether their DNA methylation might contribute to cancer (such as *2C35*), further experiments will be required to determine whether hypermethylation has functional consequences. Examining the biological consequences of sequential gene silencing, for example in AAH- or AIS-derived cell lines [104], will help confirm the role of the genes under study in lung adenocarcinoma development and progression. Further delineating the nature and timing of epigenetic hits, which are in principle reversible, is potentially highly relevant for epigenetic therapy of early lung cancer, and perhaps for cancer prevention. Lastly, irrespective of the biological effects of hypermethylation at each locus, the presence of DNA methylation characteristic of each type of lesion can be used to inform the generation of biomarkers specific for the different developmental stages of lung adenocarcinoma.

## Supporting Information

Table S1 Genes, primers and probes.
(DOC)

Table S2 Information on subjects from whom samples were obtained.
(DOC)

Table S3 Comparison between high-grade and low-grade AAH lesions.
(DOC)
