# Supplementary material for: DNA Methylation Changes in Atypical Adenomatous Hyperplasia, Adenocarcinoma In Situ, and Lung Adenocarcinoma
Source: PLoS One. 2011 Jun 23;6(6):e21443. doi: 10.1371/journal.pone.0021443 (PMC3121768; doi:10.1371/journal.pone.0021443)
Supplement: Table S1 — Genes, primers and probes. (DOC) [file pone.0021443.s001.doc]

Table S1. Genes, primers and probes^1^.

| HUGO ID^2^ | Reaction ID | Gene Name; Alternate or previous names | Forward Primer Sequence | Reverse Primer Sequence | Probe Oligo Sequence |  |
| --- | --- | --- | --- | --- | --- | --- |
| *2C35* | 2C35-M1 | Restriction Landmark Genome Scanning fragment, no known gene associated | TCGTTATTTAGGCGGTCGTTGT | ATCAACCCCATTCTTACGCTTC | CAAAACCCGCGACGCAACGAAA |  |
| *CDH13* | CDH13-M1 | Cadherin 13; H (heart)-cadherin; CDHH | AATTTCGTTCGTTTTGTGCGT | CTACCCGTACCGAACGATCC | AACGCAAAACGCGCCCGACA |  |
| *CDKN2A* | CDKN2A-M3 (exon 2 region) | cyclin-dependent kinase inhibitor 2A; p16; INK4A | GCGTTCGAGTGGCGGA | CTCCCGAACAACGTCGTACAC | CAATTAAACTCCGCGCCGTAAAACAACAA |  |
| *CDX2* | CDX2-M1 | Caudal type homeo box 2: CDX3 | GGTAATCGTCGTAGTTCGGGTATT | ACTCCGTACGCCACTCTAACG | CAACCTAACGCCGCAAAACTTCGTCA |  |
| *EYA4* | EYA4-M3 | Eyes absent homolog 4 (Drosophila):DFNA10; CMD1J | TGGATAGGATGGAAGTTTTGCG | AACTACCGACAACGCGACG | CGCTCCGACCGTTCCCGACTT |  |
| *HOXA1* | HOXA1-M1 | Homebox protein A1; HOX1F; HOX1 | GTTGTTGCGGCGATTGTAAA | CGCGCAAAACGCAACTT | TACTCTTCTTCGCTCCAACACTCCAAATCG |  |
| *HOXA11* | HOXA11-M1 | Homebox protein A11; HOX1I; HOX1 | TTTTGTTTTCGATTTTAGTCGGAAT | TAATCAAATCACCGTACAAATCGAAC | ACCACCAAACAAACACATCCACGACTTCA |  |
| *NEUROD1* | NEUROD1-M1 | Neurogenic differentiation 1; BETA2; BHF-1; NeuroD; bHLHa3; MODY6 | GTTTTTTGCGTGGGCGAAT | CCGCGCTTAACATCACTAACTAAA | CGCGCGACCACGACACGAAA |  |
| *NEUROD2* | NEUROD2-M1 | Neurogenic differentiation 2; NDRF; bHLHa1 | GGTTTGGTATAGAGGTTGGTATTTCGT | ACGAACGCCGACGTCTTC | CGCCATACGAACCGCGAAACGAATATAA |  |
| *OPCML^3^* | OPCML-M1 | Opioid binding protein/cell adhesion molecule-like; OPCM; OBCAM; IGLON1 | CGTTTCGAGGCGGTATCG | CGAACCGCCGAAATTATCAT | AACAACTCCATCCCTAACCGCCACTTTCT |  |
| *PTPRN2* | PTPRN2-M1 | Protein tyrosine phosphatase, receptor type, N polypeptide 2; KIAA0387; phogrin; ICAAR; IA-2beta | CGTTTTAATAGTTTCGGGTTTAGTTATAAGT | AACTACGCTTTCTCAACGCCTC | TAAAACGACCGCGTACTCGCCAAAAAA |  |
| *RASSF1* | RASSF1A-M1 | Ras association (RalGDS/AF-6) domain family 1; NORE2A; REH3P21; RDA32; 123F2 | ATTGAGTTGCGGGAGTTGGT | ACACGCTCCAACCGAATACG | CCCTTCCCAACGCGCCCA |  |
| *SFRP1* | SFRP1-M1 | Secreted frizzled-related protein 1; FRP; FRP-1; SARP2 | GAATTCGTTCGCGAGGGA | AAACGAACCGCACTCGTTACC | CCGTCACCGACGCGAAAACCAAT |  |
| *TMEFF2* | TMEFF2-M1 | transmembrane protein with EGF-like and two follistatin-like domains 2; TENB2; HPP1; TR; TPEF; CT120.2 | CGACGAGGAGGTGTAAGGATG | CAACGCCTAACGAACGAACC | TATAACTTCCGCGACCGCCTCCTCCT |  |
| *TWIST1* | TWIST1-M1 | Twist homolog (acrocephalosyndactyly 3; Saethre-Chotzen syndrome) (Drosophila) | GTAGCGCGGCGAACGT | AAACGCAACGAATCATAACCAAC | CCAACGCACCCAATCGCTAAACGA |  |
| *ALU* | ALU-C4 | ALU control reaction consensus | GGTTAGGTATAGTGGTTTATATTTGTAATTTTAGTA | ATTAACTAAACTAATCTTAAACTCCTAACCTCA | CCTACCTTAACCTCCC |  |
| *ALU* | ALU-M2 | Methylated Alu consensus | GCGCGGTGGTTTACGTTT | AACCGAACTAATCTCGAACTCCTAAC | AAATAATCCGCCCGCCTCGACCT |  |
| *SAT2* | SAT2-M1 | Methylated Satellite-2 Chromosome 1 | TCGAATGGAATTAATATTTAACGGAAAA | CCATTCGAATCCATTCGATAATTCT | CGATTCCATTCGATAATTCCGTTT |  |

^1^ All DNA sequences written in the 5’ to 3’ direction. ^2^ Most recent Human Genome Organization identifier (<http://www.genenames.org/>). ^3^ The OPCML MethyLight amplicon also targets the adjacent HNT CpG island.
