# Supplementary material for: DNA Methylation Changes in Atypical Adenomatous Hyperplasia, Adenocarcinoma In Situ, and Lung Adenocarcinoma
Source: PLoS One. 2011 Jun 23;6(6):e21443. doi: 10.1371/journal.pone.0021443 (PMC3121768; doi:10.1371/journal.pone.0021443)
Supplement: Table S2 — Information on subjects from whom samples were obtained. (DOC) [file pone.0021443.s002.doc]

**Table S2.** **Information on subjects from whom samples were obtained.**

|  | AdjNTL, AAH, AIS, Adenocarcinoma subjects | MetNTL subjects |
| --- | --- | --- |
| Number of subjects (n) | 63 | 30 |
| Median age^1^ | 65 | 60.5 |
| Age range | 42-80 | 35-74 |
| Gender^2^ | 32 F, 31 M | 11F, 19M |
| Subjects from whom smoking status is known | 19 | 0 |
| Confirmed smokers | 18 | unknown |
| Packyears | 20-100^3^ | unknown |
| Nonsmokers | 1 | unknown |
|  |  |  |

^1^No statistically significant difference in age between subjects providing AdjNTL, AAH, AIS and adenocarcinoma samples (ANOVA), statistically significant difference in age between AdjNTL and MetNTL subjects (p=0.0281, two-tailed t-test).

^2^No statistically significant difference in gender between any groups (ANOVA).

^3^Sixteen subjects had smoked for >20 packyears. One subject had smoked for less than 10 years, and packyears were unknown for one smoker.
