# Supplementary material for: DNA Methylation Changes in Atypical Adenomatous Hyperplasia, Adenocarcinoma In Situ, and Lung Adenocarcinoma
Source: PLoS One. 2011 Jun 23;6(6):e21443. doi: 10.1371/journal.pone.0021443 (PMC3121768; doi:10.1371/journal.pone.0021443)
Supplement: Table S3 — Comparison between high-grade and low-grade AAH lesions. (DOC) [file pone.0021443.s003.doc]

**Table S3. Comparison between high-grade and low-grade AAH lesions.**

|  | Median PMR^1^ | Median PMR^1^ | Median PMR^1^ |  |
| --- | --- | --- | --- | --- |
|  | LG | HG | Undesignated |  |
| Locus | (n=48) | (n=11) | (n=14) | p-value^2^ |
| *2C35* | 0.9 | 2.8 | 2.6 | 0.04 |
| *CDH13* | 0 | 0 | 0.0 | 0.48 |
| *CDKN2A ex2* | 10.2 | 13.9 | 9.2 | 0.56 |
| *CDX2* | 0.3 | 1.8 | 2.2 | 0.17 |
| *EYA4* | 0.3 | 0.2 | 2.1 | 0.91 |
| *HOXA1* | 0.0 | 0.5 | 0.2 | 0.16 |
| *HOXA11* | 1.4 | 3.0 | 0.9 | 0.21 |
| *NEUROD1* | 0.4 | 0.7 | 1.1 | 0.47 |
| *NEUROD2* | 1.6 | 0.6 | 1.7 | 0.01 |
| *OPCML/HNT* | 0 | 0 | 0 | 0.80 |
| *PTPRN2* | 3.2 | 2.7 | 1.9 | 0.58 |
| *RASSF1* | 0.0 | 0 | 0.0 | 0.22 |
| *SFRP1* | 0.1 | 0.1 | 0.2 | 0.81 |
| *TMEFF2* | 4.3 | 7.0 | 7.4 | 0.31 |
| *TWIST1* | 0 | 0 | 0 | 0.12 |

^1^PMR= Percentage Methylated Reference

^2^p-values are from GEE analysis of low grade versus high grade on ranked PMR values. No statistically significant p-values were seen (cutoff p<0.0033 after Bonferroni correction of 15).
